# Efficient protein structure search using indexing methods

**DOI:** 10.1186/1472-6947-13-S1-S8

**Published:** 2013-04-05

**Authors:** Sungchul Kim, Lee Sael, Hwanjo Yu

**Affiliations:** 1 POSTECH, Pohang, South Korea; 2 SUNY Korea, Incheon, South Korea

## Abstract

Understanding functions of proteins is one of the most important challenges in many studies of biological processes. The function of a protein can be predicted by analyzing the functions of structurally similar proteins, thus finding structurally similar proteins accurately and efficiently from a large set of proteins is crucial. A protein structure can be represented as a vector by 3D-Zernike Descriptor (3DZD) which compactly represents the surface shape of the protein tertiary structure. This simplified representation accelerates the searching process. However, computing the similarity of two protein structures is still computationally expensive, thus it is hard to efficiently process many simultaneous requests of structurally similar protein search. This paper proposes indexing techniques which substantially reduce the search time to find structurally similar proteins. In particular, we first exploit two indexing techniques, i.e., iDistance and iKernel, on the 3DZDs. After that, we extend the techniques to further improve the search speed for protein structures. The extended indexing techniques build and utilize an reduced index constructed from the first few attributes of 3DZDs of protein structures. To retrieve top-*k *similar structures, top-10 × *k *similar structures are first found using the reduced index, and top-*k *structures are selected among them. We also modify the indexing techniques to support *θ*-based nearest neighbor search, which returns data points less than *θ *to the query point. The results show that both iDistance and iKernel significantly enhance the searching speed. In top-*k *nearest neighbor search, the searching time is reduced 69.6%, 77%, 77.4% and 87.9%, respectively using iDistance, iKernel, the extended iDistance, and the extended iKernel. In *θ*-based nearest neighbor serach, the searching time is reduced 80%, 81%, 95.6% and 95.6% using iDistance, iKernel, the extended iDistance, and the extended iKernel, respectively.

## Introduction

The size of protein structure database such as the Protein Data Bank (PDB) continues to grow. PDB had around 1000 structures in 1992, but now it stores over 150,000 structures. In addition, the number of proteins with unknown functions is increasing due to efforts in structural genomics projects. Knowing the functions of proteins is crucial to many studies of biological processes. Especially, researchers need to know the key proteins that play an important role for severe deseases, and it is directly related to human life. Therefore, assigning functions to novel proteins is one of the most significant problems in proteomic study, and several methods have been developed to assign functions to an unknown protein. Basically, the function of a protein can be identified by searching amino acid sequence database for similar sequences that the functions are already known. However, the 3D structures of proteins are more conserved than the sequences and using the structural information provide more reliable similarity measures.

Many methods have been introduced for pair-wise protein structural search. They align the two structures and compute the Root Mean Square Deviation (RMSD) between the core atomic positions, e.g., alpha carbon coordinates, of the aligned proteins. However, most of methods based on structural alignment cannot be used to search structures against large database, since it has high computational complexity. Sael et al. introduced a new approach for fast protein surface similarity search using 3DZDs [1]. This approach does not consider individual residue/atom positions, or the arrangement of the secondary structure segments. 3DZD has three advantages: 1) fast k-nearest neighbor search, 2) rotational invariance, and 3) easy adjustment of the resolution of the structural representation resolution. In particular, using 3DZDs, it is possible to retrieve similar protein structures in seconds among 150k protein structures. However, few seconds are still too long for a real time search system, since response time increases further when multiple search requests are processed simultaneously. To enhance the searching speed, using indexing technique could be a good solution [2,3]. We exploit indexing techniques on 3DZDs in order to speed up protein structure search. Specifically, we apply two indexing techniques, iDistance and iKernel, on 3D-Surfer data set, and extend them for further speed up [4]. To fully take advantage of the indexing techniques, we also provide *θ*-based nearest neighbor search which returns data points less than *θ *to the query point. The experimental results show that the indexing techniques both decrease the searching speed, and our nearest neighbor search algorithms further speed up the protein structure search. Speicifically, in top-*k *nearest neighbor search, the searching time is reduced 69.6%, 77%, 77.4% and 87.9%, respectively using iDistance, iKernel, the extended iDistance, and the extended iKernel. In *θ*-based nearest neighbor serach, the searching time is reduced 80%, 81%, 95.6% and 95.6% using iDistance, iKernel, the extended iDistance, and the extended iKernel, respectively.

This paper is organized as follows. We briefly introduce related works about protein structure search and top-k query search. Then, we explain iDistance, iKernel and the extended top-k query search method in combination with iDistance and iKernel. Finally, we provide experimental results to verify the efficiency of our approaches, and conclusion with future works.

## Related work

### Protein structure

A protein consists of a sequence of amino acid (AA) residues. A sequence of AA residues folds into a 3-dimensional (3D) structure in space and forms a functional protein. A 3D structure of a protein is recorded in a pdb file format as a set of Cartesian coordinates of all the atoms in the protein. The 3D structure contains rich information relating to function and evolution of the protein.

### Protein structure search

Earlier structural similarity measurements were designed for pair-wise analysis where the user only needed to compare handful of protein structures [5-7]. However, as the number of known structures increased more methods are proposed for similarity search in protein database [8,9]. One of the most intuitive approaches is to compare the coordinates of corresponding residues or atoms of proteins after structural alignment [10,11]. Root Mean Square Deviation (RMSD) is often used as the similarity measure. Due to its high computational complexity, structure alignment is done by using Dynamic Programming (DP) or its extensions [6,12,13].

There are major structure databases such as PDB, CATH [14], and SCOP [15] which provides only keyword search and browsing of pre-computed classification. Some database systems that are able to take a query structure are for the search includes Distance matrix ALIgnment (DALI) server [16], Vector Alignment Search Tool (VAST) search [17], and eF-site database [18]. Given a query protein structure, they need around an hour to finish searching their databases. Zeyar et al. suggests an indexing method called ProtDex for fast search in 3D protein structure database [19]. Although it performs faster than DaliLite [8], one of the most popular protein structure search algorithms, the search time of ProtDex takes over a few minutes and it is not practical for online database searches.

### 3D-Surfer

3D-Surfer is a new and efficient protein structural search system which represents protein structures based on 3D-Zernike Descriptor (3DZD). The major advantage of 3DZD is that it allows a fast k-nearest neighbor (k-nn) search of protein structures. It has been verified that the retrieved k-nn proteins by 3D-Surfer have similar functional and evolutional information in terms of SCOP classification [20]. Some of the characteristics of the 3DZDs is that it is rotational invariant, and the resolution of the representation of protein structures are easily adjusted by changing the order, and descriptors of the lower order are contained in the descriptors of the higher order.

### Nearest neighbor search algorithm

There is a long stream of researches on finding nearest neighbor search problem which is an optimization problem for finding closest points in metric spaces. The simplest method is to compute the distance from the query point to every other point in the database. It has *O*(*Nd*) complexity where *N *is the number of data points and *d *is the dimensionality of the data, and 3D-Surfer also used this approach. For efficient top-k search, there have been various methods via space partitioning including X-tree, TV-tree, and SR-trees [21-23]. iDistance that we used here is also space partitioning method. There are other methods such as iKernel which is an indexing technique and designed for efficient calculation of support vector machine (SVM). The details of iDistance and iKernel are described in the methods. Note that those methods cannot be directly used for protein structure data, thus in this work we exploit the 3DZD of protein structures and apply indexing techniques on the 3DZDs.

## Methods

In this section, we first introduce the protein structure dataset and their 3D-Zernike Descriptor (3DZD). Then, the descriptions of iDistance and iKernel methods and the proposed efficient top-k query search method based on the characteristics of 3DZD are provided.

### Protein structural dataset and 3D-Zernike Descriptor

3DZDs are compact and rotationally invariant representation of 3D structures. 3DZD has been successfully used for protein [1] and ligand structure analyses [24] as well. We provide brief description of 3DZD for reader's convenience. Detailed description can be found in [25,26].

The 3DZD descriptors for protein structural dataset of 158781 number of protein chain structures was obtained through 3D-Surfer database. The entire structures in PDB was collected and processed on 2009 [27]. For each of the pdb files that contain one to several protein chains, the chains were separated and surfaces of each chain were obtained through molecular surface calculation program, MSROLL version 3.9.3 [28], and then voxelized. Each of the voxelized protein surface were used as a input to 3DZD conversion program and a vector of 121 numbers called invariants were computed.

In 3DZD construction, a given 3D function *f*(*x*) that contains a surface information of protein is expanded into a series of Zernike-Canterakis bases defined as follows:

(1) $$Z_{nl}^{m}\left(r,\;\;\vartheta ,\;\;\varphi \right)=R_{nl}\left(r\right)Y_{l}^{m}\left(\vartheta ,\;\;\varphi \right)$$

where *-l < m < l*, 0 ≤ *l *≤ *n*, (*n - l*) is even, $Y_{l}^{m}\left(\vartheta \text{,}\;\;\varphi \right)$ are spherical harmonics, and *R_nl _*are radial functions constructed to convert $Z_{nl}^{m}\left(r,\;\;\vartheta ,\;\;\varphi \right)$ to polynomials in the Cartesian coordinates, $Z_{nl}^{m}\left(x\right)$. To obtain the 3DZD of *f*(*x*), 3D Zernike moments need to be computed first. They are defined by expanding the orthonormal bases as follows:

(2) $$\Omega_{nl}^{m}=\frac{3}{4\pi }\int_{|x|\leq 1}f\left(x\right){\bar{Z}}_{nl}^{m}\left(x\right)dx$$

Then, the 3DZD, *F_nl_*, is computed by normalizing $\Omega_{nl}^{m}$ as follows:

(3) $$F_{nl}=\sqrt{\overset{m=l}{\underset{m=-l}{\sum }}{\left(\Omega_{nl}^{m}\right)}^{2}}$$

where *n *is the order of 3DZD determining the resolution of the descriptor. Then, the norms allow ratational invariance to the desriptor. For each pair of *n *and *l*, 3DZD has a series of invariants, the numbers in the vector of 3DZD, where *n *is ranged from 0 to the predefined order (20 in this case).

**Figure 1:**
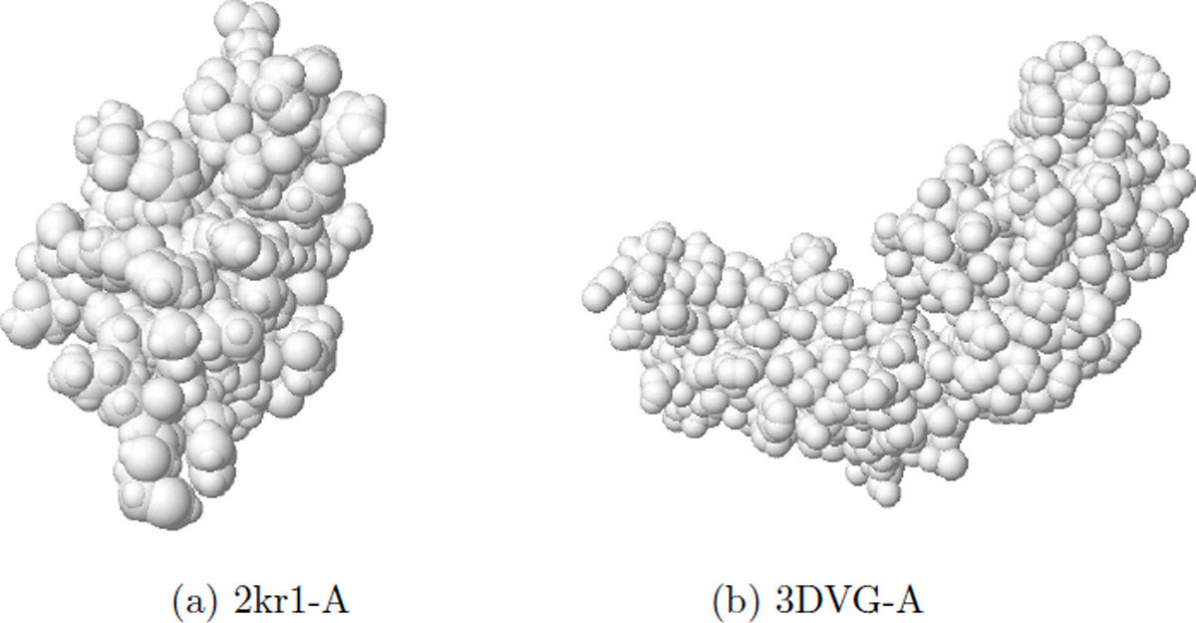
**Two example proteins**. (a) 2kr1-A and (b) 3DVG-A.

**Figure 2:**
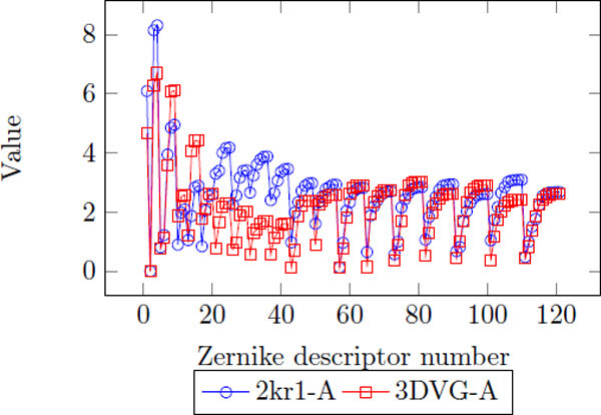
**3D Zernike descriptors of two example proteins**. The dimension of 3DZD is 121.

Figure 1 is an example of 3DZD of two proteins, triosephosphate isomerase (PDB code: 2kr1-A) and interleukin-4 receptor *α*-chain (PDB code: 3DVG-A). As you can see, two proteins have different structures overall and their descriptor also shows visible difference (Figure 2).

### Indexing techniques

In this work, we exploit two indexing techniques: iDistance and iKernel. Two indexing techniques partition given data points into clusters and using the clusters to find k-nn. Note that both techniques exactly retrieve k nearest neighbor results given a query. The details of the two indexing methods follows.

#### iDistance

iDistance is an efficient indexing technique for k-nearest neighbor search in a high-dimensional metric space [29]. It depends on how data are partitioned and how reference points for each partition are defined (we will henceforth mention partition as cluster for terminology consistency between iDistance and iKernel). After clustering and reference point selection, each data point is indexed according to the distance between its reference points.

To build an index, the reference points are selected by data clustering. Although various clustering techniques can be used to select reference points, we have used k-means clustering. And then, data points are assigned its closest reference point. During the assignment process, the data point is recorded with the distance which is called iDistance and is used as a key for top-k search. The iDistance is computed as follows:

(4) $$y=i\times C+dist\left(p,O_{i}\right)$$

where *y *is iDistance of point *p *in *i*-th cluster, *O_i_*, and *C *is a constant used to stretch the data ranges of indexes.

**Figure 3:**
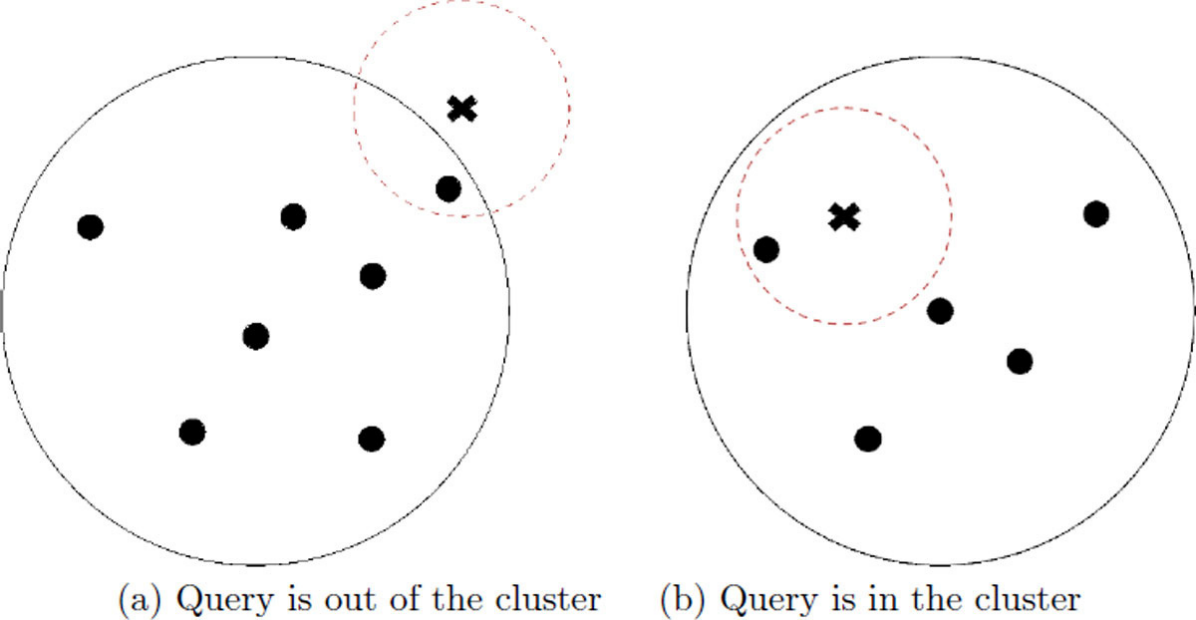
**Top-k search using iDistance**. x mark is query; circle mark is data point; circle with solid line is cluster; circle with dashed line is query region.

To retrieve top-k results, we visit the clusters to check whether the cluster can have nn or not. The radius *r *that indicates query region defined as the range from the query, and *r *increases by Δ*r *to form a larger query region after iterations. When the query region is overlapped with certain cluster, we notice that the cluster will have nearest points. Therefore, at each iteration, we first check whether the target cluster *C_i _*can have nn of query *q *by comparing the distance from *q *to the reference point of *C_i_*, and the farthest distance in *C_i_*. If the area of *C_i _*overlaps with the query region (Figure 3-(a)), it indicates that *C_i _*can have nearest neighbor. Therefore, we check the data points in the cluster to find the nearest points from the outermost position of the cluster. If *q *is located in *C_i _*(Figure 3-(b)), it also indicates that *C_i _*have nn. In this case, we need to search the cluster inward and outward from the position of *q*.

#### iKernel

iKernel is originally designed for the efficient learning of support vector machine (SVM) [30]. However, it is also applicable for top-k search for Euclidean distance. Similar to iDistance, it first divide given data points to clusters where the clusters have set of rings which are the data structure defined for iKernel. Given a query, it searches k-nn by visiting each cluster and its rings.

To build an index, given data points are clustered into *m *clusters and centroids of each clusters are computed in the feature space. Various clustering techniques can be applied. Then, based on those centroids and clusters, we can build an index by assigning data points into a set of rings in clusters as follows.

(5) $$\begin{array}{cc} C_{1} & :\left\{C_{1,1}:x_{1,1}^{\left(1\right)},x_{1,1}^{\left(2\right)},\cdots \;,x_{1.1}^{\left(g\right)}\right\},\left\{C_{1,2}:x_{1,2}^{\left(1\right)},x_{1,2}^{\left(2\right)},\cdots \;,x_{1,2}^{\left(g\right)}\right\},\cdots \\ C_{2} & :\left\{C_{2,1}:x_{2,1}^{\left(1\right)},x_{2,1}^{\left(2\right)},\cdots \;,x_{2.1}^{\left(g\right)}\right\},\left\{C_{2,2}:x_{2,2}^{\left(1\right)},x_{2,2}^{\left(2\right)},\cdots \;,x_{2,2}^{\left(g\right)}\right\},\cdots \\ \cdots \; & \end{array}$$

Note that each ring have *g *number of data points. The paramter *g *is user adjustable and need to be determined prior to index construction.

To process a k-nn of a query structure, we exploits the index and Minimal Possible Distance (MPD) [30]. MPD is the minimal possible distance between a query *q *and ring structure *C_i,j_*. With this new notion of the MPD, k-nn search works as follows. Given a query point *q*, we first initialize a priority queue *Q *with a set of pair ¡*C_i,j_*, MPD¿ of each cluster in the ascending order of their MPDs between the *q *where only the outermost ring is considered first. Then, at each iteration, the top entry of *Q *is popped. If a ring is popped, the data points in the ring are inserted to *Q *with the distance from *q*, and if the popped item is a data point, it is simply added into top-k result since the priority queue ensures that all instances in the queue have larger distances from *q *and also all rings have larger MPDs between *q*.

**Figure 4:**
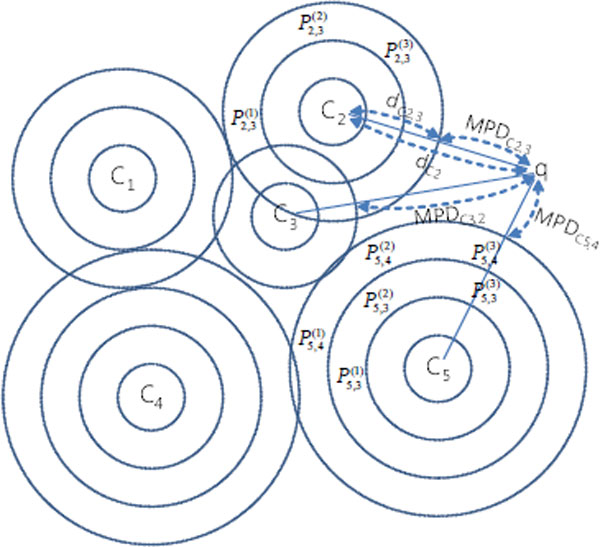
**Top-2 query processing in iKernel**. q is query point, *C_i _*is i-th cluster, there are same number of data points in rings $\left(P_{i,j}^{n}\right)$, and *MPD_i,j _*is the mininum possible distance of j-th ring of i-th cluster.

We now explain how top-k processing can be done with an example (Figure 4 and Table 1) when k = 2. In the queue, *Q*, rings or data points are ordered in the distance in ascending order (In the table, the leftmost item has the lowest distance). At the first iteration, *C*_5,4 _with the lowest MPD is popped. Then the distance from q to its instances, $P_{5,4}^{\left(1\right)}$, $P_{5,4}^{\left(2\right)}$, and $P_{5,4}^{\left(3\right)}$, and the MPD to its inner ring *C*_5,3_, are computed and these instances are inserted back to Q (the second row in Table 1). At the second iteration, the top instance $P_{5,4}^{\left(3\right)}$ is added to output since it is guaranteed to be top-k. Then, the priority queue ensures that all data points in the queue are farther from *q*. and all rings have larger MPDs to *q *which suggests that all the data points within these rings have larger distance to *q *as well. At the third iteration, the top instance, ring *C*_5,3 _is popped and its data points $P_{5,3}^{\left(1\right)}$, $P_{5,3}^{\left(2\right)}$, and $P_{5,3}^{\left(3\right)}$ are added to the queue. At the fourth iteration, the top instance, a ring *C*_2,3 _is popped and its instances $P_{2,3}^{\left(1\right)}$, $P_{2,3}^{\left(2\right)}$, and $P_{2,3}^{\left(3\right)}$ are added to the queue. Lastly, the top instance, $P_{5,3}^{\left(3\right)}$ is popped and returned as output. Then the top-k processing terminates since top k = 2 instances are identified.

**Table 1:** Top-2 query processing

step	output	top	**updated Q (new items in bold)**
0			*C*_5,4_, *C*_2,3_, *C*_3,2_, *C*_1,3_, *C*_4,4_
1		*C*_5,4_	$P_{5,4}^{\left(3\right)}$, C_5,3_, *C*_2,3_, $P_{5,4}^{\left(2\right)}$, $P_{5,4}^{\left(1\right)}$, *C*_3,2_, *C*_1,3_, *C*_4,4_
2	$P_{5,4}^{\left(3\right)}$	$P_{5,4}^{\left(3\right)}$	*C*_5,3_, *C*_2,3_, $P_{5,4}^{\left(2\right)}$, $P_{5,4}^{\left(1\right)}$, *C*_3,2_, *C*_1,3_, *C*_4,4_
3	$P_{5,4}^{\left(3\right)}$	*C*_5,3_	*C*_2,3_, $P_{5,3}^{\left(3\right)}$**C_5_**,**_2_**, $P_{5,4}^{\left(2\right)}$, $P_{5,3}^{\left(2\right)}$, $P_{5,4}^{\left(1\right)}$, $P_{5,3}^{\left(1\right)}$, *C*_3,2_, *C*_1,3_, *C*_4,4_
4	$P_{5,4}^{\left(3\right)}$	*C*_2,3_	$P_{5,3}^{\left(3\right)}$, *C*_5,2_, $P_{5,4}^{\left(2\right)}$, **C_2_**,**_2_**, $P_{5,3}^{\left(2\right)}$, $P_{2,3}^{\left(3\right)}$, $P_{5,4}^{\left(1\right)}$, $P_{5,3}^{\left(1\right)}$, $P_{2,3}^{\left(2\right)}$, $P_{2,3}^{\left(1\right)}$, *C*_3,2_, *C*_1,3_, *C*_4,4_
5	$P_{5,4}^{\left(3\right)}$, $P_{5,3}^{\left(3\right)}$	$P_{5,3}^{\left(3\right)}$	*C*_5,2_, $P_{5,4}^{\left(2\right)}$, *C*_2,2_, $P_{5,3}^{\left(2\right)}$, $P_{2,3}^{\left(3\right)}$, $P_{5,4}^{\left(1\right)}$, $P_{5,3}^{\left(1\right)}$, $P_{2,3}^{\left(2\right)}$, $P_{2,3}^{\left(1\right)}$, *C*_3,2_, *C*_1,3_, *C*_4,4_

#### Partitioning

For both indexing techniques, we require division of data set into clusters by assigning similar number of data points to each cluster. First method randomly selects reference points and assign remaining data points to their closest reference point. K-means clustering, which is one of the most widely used clustering methods, is also tested. The goal of K-means clustering algorithm is to divide a set of points into *k *clusters so that the within-cluster sum of squares is minimized [31]. K-means algorithm is easily applicable to problems and performance is often shown to be satisfying. However, it also has some disadvantages as the K-means algorithms is a local search procedure and it suffers from the serious drawback that its performance depends on the initial starting conditions [32]. Therefore, in this work, we repeatedly cluster data points and conduct experiments, and select the best result.

**Figure 5:**
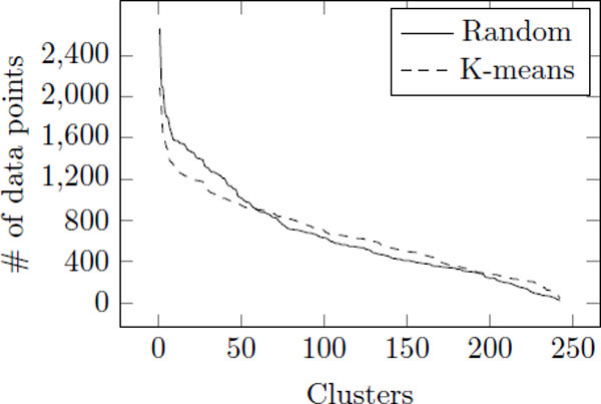
**The number of data instances assigned to clusters by random and k-means clustering**. The solid line is random clustering, and the dashed line is K-means clustering.

Figure 5 shows that the number of data points assigned to clusters. As you can see, using K-means generates more stable clusters with similar amount of data points. If some clusters have larger number of data points than other clusters, we need to search their region more than other clusters. Therefore, balancing the number of data points in clusters is required for efficient k-nn search. In experiment, we compare those partitioning strategies in terms of search speed.

### Extended top-k search based on 3DZD

**Figure 6:**
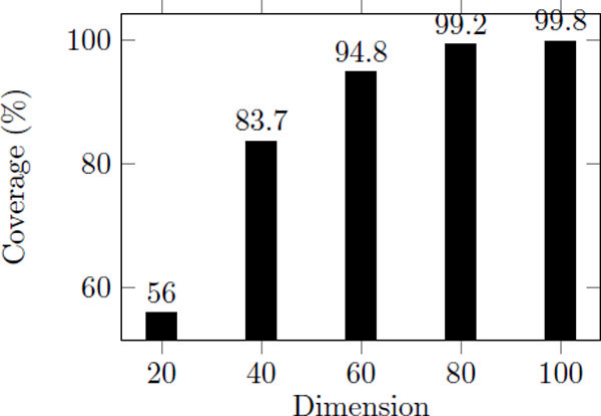
**The coverage of top-25 in top-250 as the dimension used for index increases**. The number of clusters, *M*, is 866, *<*Δ*r*, *C >*is *<*0.2, 4 *>*, and the number of data points in each ring, *g *is 50.

**Figure 7:**
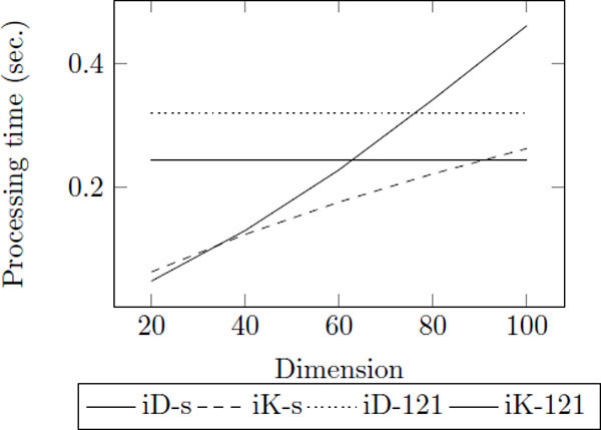
**The change of processing time as the dimension used for index increases**. The number of clusters, *M*, is 866, *<*Δ*r*, *C >*is *<*0.2, 4 *>*, and the number of data points in each ring, *g *is 50.

According to 3DZD, the descriptor vector has one notable characteristic. The prior dimensions in the descriptor vector indicate global shape and the posterior dimensions include more specific shape information. Based on this fact, we propose an extended top-k search approach for protein structure based on 3DZD. In this approach, the index is constructed based on the data set using only the prior half of original vectors. Retrieval result of top-250 using first 60 invariants in the descriptor vectors covered 94.8% of the top-25 retrieval result using the full descriptors as shows in Figure 6. In addition, finding top-*k *× 10 result using half dimension takes less time than using basic indexing techniques (Figure 7) where iD-s and iK-s is the result of top-*k *× 10 for iDistance and iKernel using small dimensions, respectively, and iD-121 and iK-121 is the result of top-*k *for iDistance and iKernel using original 121 dimensions. This shows that using half of the descriptor for indexing allows a fast and accurate approximation of using the full descriptor.

Based on this observation, we introduce a new approach for top-k search as follows.

1 Given a query protein Q, search top-*k *× 10 result using the indexing structure with 60-dim (the half of entire dimension).

2 Using the top-*k *× 10 results, find exact top-*k *result.

Note that *k *× 10 is very small number compared to the size of the database (around 1.6 million).

### Threshold-based nearest neighbor search

**Figure 8:**
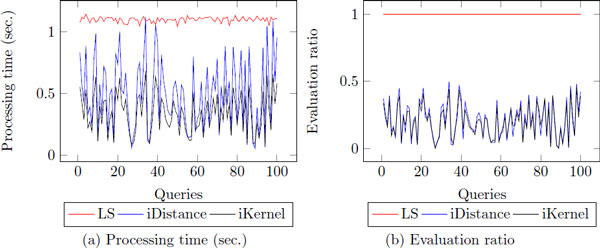
**The distribution of processing time of fKNNs**. The experiment is conducted with 100 randomly selected query proteins.

In preliminary experiments, we found that the processing time/evaluation ratio of the top-k nearest neighbor search is very different depending on the queries. It is different from the linear search which always shows stability as *q *varies (Figure 8). Intuitively, we can see that if the distance between *k*-th nearest neighbor and query is large, both indexing techniques need to visit more clusters and the list of *k*-th nearest neighbor is frequently changed during the search procedure. In addition, if the distance between two proteins is large, it indicates that they are not similar in terms of their structural information and they do not share functional information as well. Therefore, we cast the nearest neighbor search task as threshold-based nearest search in order to guarantee stable processing time/evaluation ratio with reliable results.

The nearest neighbor search can be solved based on two different user parameters of either the number of nearest neighbor, *k*, or the threshold of the distance between nearest neighbors and query, *θ *(From now on, we call the second approach as *θ*-based nearest neighbor seach). Therefore, we exploit the indexing techniques in *θ*-based nn search as well. Using *θ*, a data point can be nearest neighbor, only if they have shorter distance to the query than *θ*. To do this, in the linear scan, we need to check whether the distance between each protein structure and query is less than *θ *or not, we do not need to check the number of nearest neighbor, *k*. In the iDistance, we set the query range, *r *as *θ*. Then the number of visiting clusters and computing the distance between their data points ot query decreases, when the number of nearest neighbor with shorter distance than *θ *has smaller than *k*. In the iKernel, the search process is terminated untill the Minimum Possible Ditance (MPD) of the popped instance is smaller than *θ*. It also reduces the cost of visiting clusters and computing the distance bewteen the query and their data points. Note that, using *θ*, the extended approaches always return the exact nearest neighbors.

## Results

In this section, we verify the effectiveness of indexing techniques on top-k search of protein structures. Sael et al. showed that 3DZD works well on finding similar proteins in terms of functional and evolutionary characteristics based on SCOP classification [1]. The SCOP provides the ordering of all proteins of known structure according to their evolutionary and structural relationships. In addition, both of iDistance and iKernel are not approximate techniques and find exact top-k nn from database according to the structural similarity described by 3DZD. Therefore, we only measure the efficiency in terms of processing time and evaluation ratio. The evaluation ratio is computed as the fraction of accessed data points over the number of database (1 for linear scan since it access all data points in the data set). The processing time could be affected by the various factors including performance of machine, the number of users, and network environment. In contrast, the evaluation ratio shows consistent measure.

The experiments were conducted on the machine, Intel Core(TM) i7 CPU (3.40GHz), and 16 GB memory. In overall experiment, we used 100 data points that are randomly selected from data set, and averaged entire processing time and evaluation ratio.

### The user parameters

There are a few parameters that are needed to be optimized in the iDistance and iKernel methods. In this section, we observe how the result varies as the user parameters varies to select the best. We also observe how the cluster number affects the top-k search. We vary the partition data points using different number of clusters: 121, 242, 498, and 866. 121 is the dimensionality of data set, and 242 is the two times the dimensionality ([29] refers that this way works well on iDistance). And the others are according to SCOP classification hierarchy. 498 is the number of families, and 866 is the number of protein domains [15]. We assume that the numbers defined by domain experts could have good evidence of cluster number. The followings include the explanation of user parameters and their experimental result.

**Figure 9:**
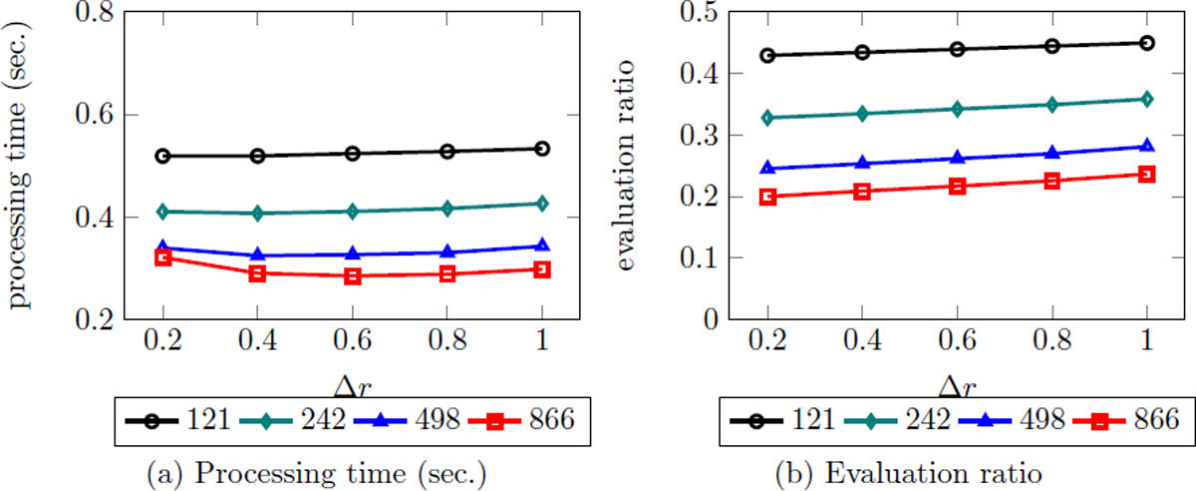
**The efficiency of top-k search using iDistance with various Δ*r***. Δ*r *is the amount of value added to *r *after each iteration in iDistance.

There are two user paramters: Δ*r *and *C *in iDistance. *r *is the distance radius of query region that indicates an area that we need to search, and Δ*r *is the amount of value added to *r *after each iteration, and *C *is used to obtain key value for index construction. Although we have conducted some experiments to tune *C *as well, it seems not affect much on top-k search. Therefore, in this work, we set *C *as 4 by maintaining few of data instances are overlapped between clusters as [29] did. Figure 9 shows the changes of processing time and evaluation ratio as Δ*r *increases. The results show that smaller Δ*r *and larger number of clusters generally performs better. It indicates that iDistance depends highly on the number of clusters. In contrast to statement made by Jagadish et al. that using two times the dimension of data as the number of clusters often works well, the result show that when the data size is very large, large number of clusters is needed as well. Therefore, different from Jagadish's work [29], it is likely that when the data size is very large, we have to use large cluster number as well.

For the optimized value, we decided to use 0.2 as Δ*r *and 866 as the number of clusters, since it shows the best result in terms of the evaluation ratio. Though it does not result out the best result in terms of processing time, the difference among Δ*r *is not that large compared to the difference among the dimensions.

**Figure 10:**
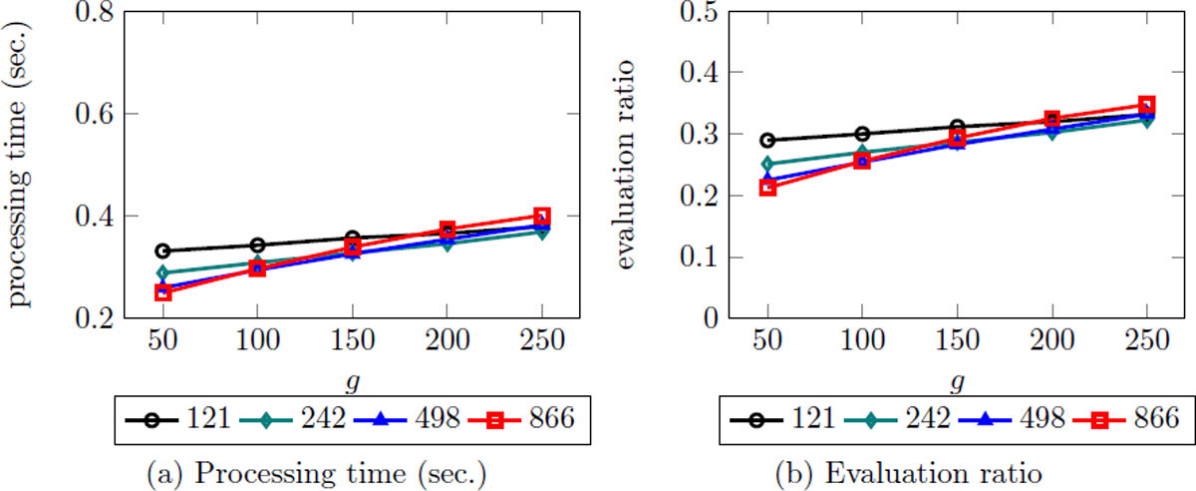
**The efficiency of top-k search using iKernel with *g***. *g *is the number of data points in each ring when iKernel is used.

There is one parameter, *g*, in iKernel. The parameter *g *is the number of data points in rings of the clusters. As mentioned before, for top-k search, we visit the rings according to its MPD and add all data instances of the ring into the priority queue, which is sorted at the end of every iteration. Figure 10 shows that the best performance is obtained using the 50 when the number of cluster is 866. The result also seems that when the number of cluster is small, the amount of changes as *g *varies becomes small. When the number of clusters is small, the number of rings decreases as well, so that the result affects by *g *less than when the number of clusters is large.

For the optimized value, we decided to use 50 as *g *and 866 as the number of clusters, since it shows the best result in terms of the evaluation ratio as well as the processing time.

### The comparison of clustering techniques

First, we compare the performance of the two clustering approaches: Random clustering and K-means clustering algorithm. In addition to normal clustering requirement of high inter-distance and low intradistance between the clusters, for efficient indexing purposes, we require that the size of clusters are balanced, that is clusters should have similar number of data points. Intuitively, if some clusters have more data points than others, search time for those clusters will be high. Table 2 shows that indexing techniques with k-means clustering, although slightly slower, have better evaluation ratio than random clustering. Therefore, the following results in later sections use the result by the indexing techniques with k-means algorithm.

**Table 2:** The effectiveness of clustering (Processing time (sec.)/Evaluation ratio)

	iDistance	iKernel
Random	0.34124/0.2132	0.26173/0.2251
K-means	**0.3237**/**0.2**	**0.246**/**0.2122**

### The number of nearest neighbor, k

**Figure 11:**
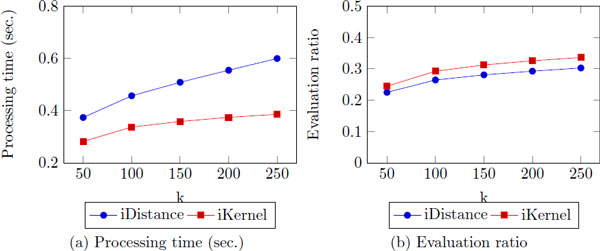
**The processing time/evaluation ratio as k increases**.

Although we have fixed the *k *to 25 in the previous experiments, we explore the effect of *k *and the performance. As expected, the processing time increases (Figure 11). However, the increase processing time is less for iKernel than iDistance as *k *increases, and iKernel shows works faster than overall. In terms of the evaluation ratio, the result is different. Though the difference is small, iDistance shows better result than iKernel. It indicates that when *k *becomes large, iKernel needs to search more data points than iDistance since it adds all of data points in visited rings computing the distance between the data point and query. However, the overall processing cost is less for iKernel.

### The filtering threshold, ***θ***

**Figure 12:**
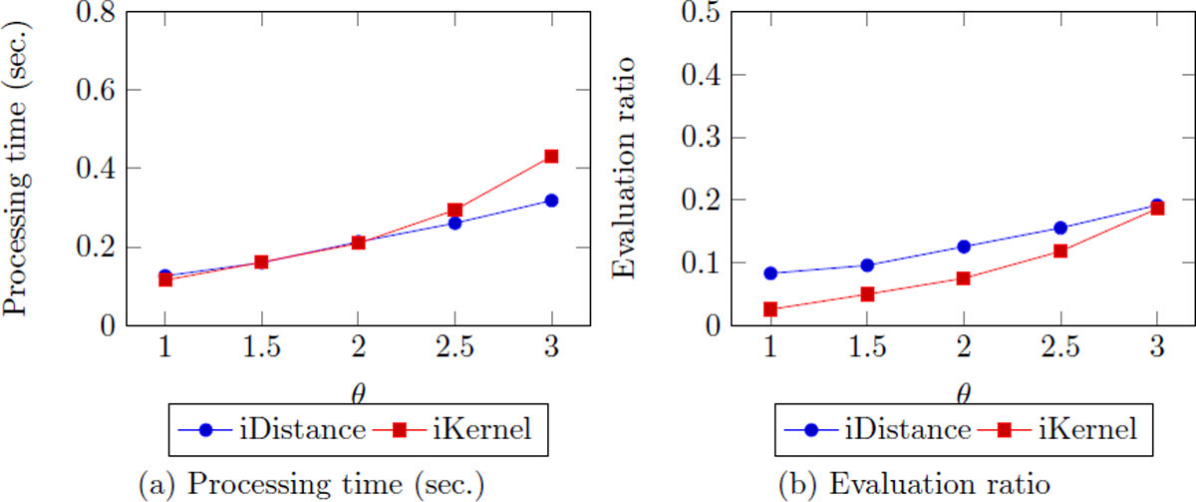
**The processing time/evaluation ratio as ***θ ***increases**.

In this section, we explore the effect of *θ *and the performance. As *θ *increases, the processing time/evaluation ratio increases as well since we need to visit more clusters and data points (Figure 12). However, still we can guarantee better and stable efficiency with exact results. Different from the result of *k*, iDistance works faster than iKernel in many cases, even its evaluation ratio is larger than iKernel. It shows that iDistance is more rubost to *θ *than iKernel. When we use *k *as nn constraint, iKernel visits less number of data points and is terminated quickly since data points divided into rings which is more specific data structure than clusters. However, when we use *θ *as nn constraint, iKernel visits more rings (and data points) than iDistance due to the same reason.

**Figure 13:**
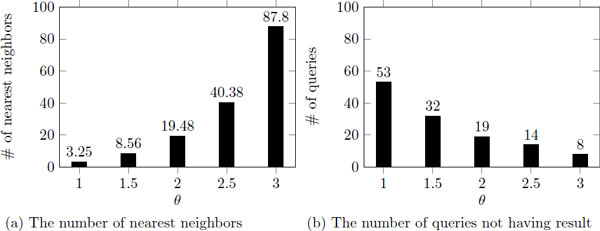
**The analysis of ***θ***-based nearest neighbor search**. The number of nearest neighbors and the number of queries without nearest neighbors as *θ *increases.

We also provide the ratio of queries not having nearest neighbors according to given queries, and the average number of nearest neighbors resulted from *θ*-based nearest neighbor search (Figure 13). As easily expected, there are more nearest neighbors and less queries with no nearest neighbors as *θ *increases. When we set *θ *as 2, the number of nearest neighbor is reasonable (similar to 25), and about 20% of queries do not have results. We use 2 as *θ *for next experiments, since the processing time/evaluation ratio is reduced enough compared to the linear search and the nearest neighbors are close to the queries compared to top-*k *nn search.

### The stability of processing time/evaluation ratio

**Figure 14:**
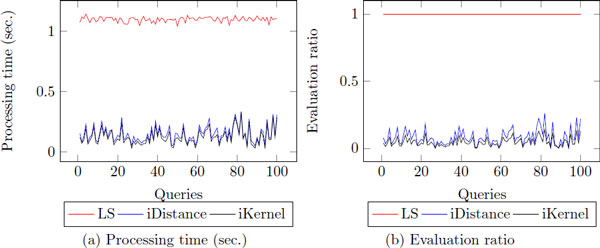
**The distribution of processing time of fTNNs**.

To show the stability of the *θ*-based nearest neighbor search, we observe the distribution of the processing time/evaluation ratio of the proposed methods. As shown before (Figure 8), when we find top-k nearest neighbors, the processing time/evaluation ratio is not stable according to different queries. According to the Figure 14, *θ*-based nn search shows more stable performance than the top-*k *nn search since using *θ *can filter out many clusters and data points which have long distance. In addition, it always retrieves exact nearest neighbors having the distance to query less than *θ*. Therefore, *θ*-based nearest neighbor search will be useful to some users who want to find nearest neighbors with tighten similarity to query protein.

### The enhancement with the extended nearest neighbor search

In this section, the result of the extended approaches is compared to the best results of the basic approach discussed in the previous section. Since we use optimal paramters based on previous experiments. First, we provide the result of the proposed approaches in top-*k *nearest neighbor problem (Table-3). In the table, the number in bracket is the ratio of actual top-25 result in top-25 result which are approximately obtained by the extended approach (which is same to the preliminary result, Figure 6). As you can see, the enhancement of iDistance and iKernel with basic top-*k *search is not that large, the extended approaches work much faster than basic approaches. In addition, iKernel always works faster than iDistance. Among iKernel results, it looks like the basic approach works faster than the extension, but it is not. Note that when they access to data instance to compute inner products of vectors, in the case of basic approach, there are 121-dimensional vectors. However, in the case of the extended approach, the inner product takes 60-dimensional vectors. It indicates that if the difference is small between two approaches, the extended approach may work better than the basic approach in real. In addition, when the number of query is small, the quality is comparable. However, when the number of query becomes large, the difference of processing time becomes larger as well.

Next, we conducted same experiments for the proposed approaches in *θ*-based nearest neighbor search (Table 4). Similar to Table 3, using index techniques works faster than the linear scan, and the extended approaches work much faster than the basic approaches. However, in *θ*-based nearest neighbor search, iDistance works faster than iKernel in many cases. The reason is described in Section. Accordingly, we conclude that iDistance is proper to find nearest neighbors which have shorter distance than the fixed distance, and iKernel is proper to find exact number of nearest neighbors. Generally, in addition, the results show that the extended approaches further speed up the basic approaches in both of top-*k *and *θ*-based nearest neighbor search on the protein structure data set.

**Table 3:** The comparison of the proposed approaches in top-*k *nearest neighbor search (Proc. is processing time measured in second and Eval. is evaluation ratio)

	LS	iDistance		iKernel	
		
		basic	**ext**.	basic	**ext**.
Proc.	1.0567	0.3212	0.2387 (95.2)	0.2434	**0.128 (95.2)**
Eval.	1	0.3	0.202 (95.2)	**0.1761**	0.1993 (95.2)

**Table 4:** The comparison of the proposed approaches in *θ*-based nearest neighbor search (Proc. is processing time measured in second and Eval. is evaluation ratio)

	LS	iDistance		iKernel	
		
		basic	**ext**.	basic	**ext**.
Proc.	1.08	0.2128	**0.0479**	0.2091	0.087
Eval.	1	0.1254	0.0537	0.075	**0.0222**

### Simulation result

**Figure 15:**
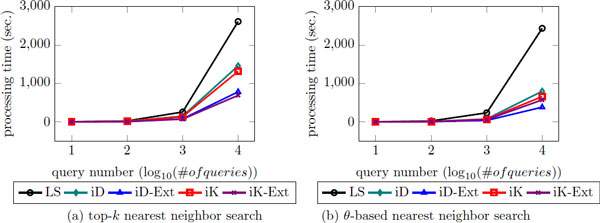
**The simulation result as the number of query increases**. x-axis is the number of users in log scale.

To support our statement more, we simulate the scenario that a number of users enter queries at the same time via multi-threading (Figure 15). According to the result, the basic approaches work faster than the linear scan and the extended approaches further speed up the basic approaches, and the *θ*-based nn search works much faster than the top-*k *nn search since using *θ *we can filter out more clusters (or rings) and data points. Using *k*, iKernel works faster than iDistance which indicates that iKernel is more efficient to find exact number of nearest neighbor with new structure, ring. In constrast, however, using *θ*, iDistance works faster than iKernel which indicates that iDistance is more appropriate to find the nearest neighbor within the fixed distance, *θ*.

## Conclusion

In this paper, we introduce an efficient indexing for protein structure search where protein structures are represented as vectors by 3D-Zernike Descriptor (3DZD). When we retrieve top-*k *nearest neighbors, using indexing techniques alone, we were able to make the search speed 77% faster compared to the prevoius version of 3D-Surfer that uses linear Euclidian distance scan between the 3DZDs in the database. We also proposed an extended version of the protein structure search based on the key observation that the prior dimension of the descriptor indicates global shape of the protein structure. Using the extended techniques it is improved up to 87.9%. When we retrieve nearest neighbor with shorter distance to the query than *θ*, using indexing techniques alone, we were able to make the search speed 81% faster compared to the linear scan. Using the extended techniques it is improved up to 96%. For future work, we will improve the nearest neighbor search with indexing techniques by utilizing the characteristics of the query prior to searching. In addition, we will apply indexing techniques for protein binding site similarity search with other data set represented based on 3DZD as well.

## Competing interests

The authors declare that there are no competing interests.

## Authors' contributions

Sungchul Kim designed models, conducted data analysis and experiments, and wrote the paper; Sael Lee designed the study, gave technical support, and wrote the paper; Hwanjo Yu gave conceptual advice, designed experiments and wrote the paper; All authors discussed the results and commented on the manuscript at all stages.

## Acknowledgements

This work was partially supported by Mid-career Researcher Program through NRF grant funded by the MEST (No. KRF-2011-0016029). This work was also supported by IT Consilience Creative Program of MKE and NIPA (C1515-1121-0003)

This work is based on an earlier work: “Indexing methods for efficient protein 3D surface search”, in *Proceedings of the ACM Sixth International Workshop on Data and Text Mining in Biomedical Informatics*, 2012 © ACM, 2012. http://doi.acm.org/10.1145/2390068.2390078

## Declarations

The publication costs for this article were funded by the corresponding author.

This article has been published as part of *BMC Medical Informatics and Decision Making *Volume 13 Supplement 1, 2013: Proceedings of the ACM Sixth International Workshop on Data and Text Mining in Biomedical Informatics (DTMBio 2012). The full contents of the supplement are available online at http://www.biomedcentral.com/bmcmedinformdecismak/supplements/13/S1.
